# Dermatophyte Infections in Children: A Cross-Sectional Study at a Tertiary Care Hospital

**DOI:** 10.7759/cureus.84078

**Published:** 2025-05-14

**Authors:** Anaswara Sree, Arun Inamadar, Annapurna G Sajjan

**Affiliations:** 1 Dermatology, Shri B M Patil Medical College and Research Centre, BLDE (Deemed to be University), Vijayapura, IND; 2 Microbiology, Shri B M Patil Medical College and Research Centre, BLDE (Deemed to be University), Vijayapura, IND

**Keywords:** clinico-epidemilogical profile, dermatophytes, dermatophytosis, tinea corporis, tinea infections, trichophyton mentagrophytes

## Abstract

Background

The prevalence of dermatophyte infections has recently surged worldwide, particularly in tropical nations like India. This is associated with changes in the clinical pattern and mycological profile among the pediatric population. This clinico-epidemiologic study aims to clarify the determinants, clinical trends, and health burden of dermatophytosis in the pediatric population.

Methodology

A total of 153 children below 15 years of age clinically diagnosed with dermatophytosis who visited the Dermatology Outpatient Department of BLDE (Deemed to be University), Shri B M Patil Medical College Hospital and Research Centre, Vijayapura, from May 2023 to January 2025 were included in this cross-sectional study. Detailed history regarding lesion duration, previous medication and consultation, personal history, and family history was documented. Skin scraping, hair, or nail clippings were taken from the lesion as specimens for microbiological studies. A direct microscopic examination of potassium hydroxide (KOH) mount and fungal culture was done in each case.

Results

The most commonly affected age group in the present study was 13-15 years. The most frequent clinical diagnosis was tinea corporis, which was followed by tinea capitis. The most prevalent manifestation of the combined form of dermatophytosis was tinea corporis with tinea capitis. Laboratory analysis revealed microscopy of a 10% KOH mount demonstrated fungal hyphae in 124 (81%) patient samples, and culture positivity for dermatophytes was present in 78 samples (51%). *Trichophyton mentagrophytes *was the most commonly isolated species, isolated from 49 patient samples (62.8%), followed by *Trichophyton rubrum *in 18 patient samples (23.1%).

Conclusion

The most common fungal species isolated was *Trichophyton mentagrophytes*, followed by *Trichophyton rubrum. *Our study highlights a shift in the clinical presentation of dermatophytosis among children, characterized by larger, more extensive lesions.

## Introduction

The most common skin infections in children are superficial mycotic infections, and their prevalence is rising [1,2,3]. Superficial dermatophytosis of glabrous skin is a significant public health issue due to the rise in chronic, recurring, resistant, and steroid-modified, challenging-to-treat tinea in recent years. Factors such as hot and humid climate, poor hygiene, and socioeconomic factors are contributing to the high burden of fungal infection [4]. Dermatophyte infections spread via direct contact, shared personal items, or contaminated surfaces [5,6].

There is a significant gap in the published epidemiological, clinical, and laboratory studies in this area, despite the fact that the problem of recalcitrant dermatophytosis is rapidly expanding in India [7,8]. The present study was done in children to evaluate the clinico-mycological profile and correlate the results of fungal cultures and KOH mounts with the clinical profile of these patients.

## Materials and methods

Source of data

Children below 15 years of age with clinically diagnosed dermatophytic infection attending the outpatient Department of Dermatology, Venereology, and Leprosy of BLDE (Deemed to be University), Shri B M Patil Medical College Hospital and Research Centre, Vijayapura, were enrolled for the study. The study was conducted from May 2023 to January 2025 with a sample size of 153 patients.

​​​​Data collection

A detailed clinical history regarding the duration of the disease, history of recurrence and type of lesion, similar complaints in the family members, and contacts with animals was recorded in all the cases. A clinical examination of the patients was done to determine the clinical type of dermatophytosis based on the site of involvement after obtaining informed consent for the study from the parents.

Sample collection

The affected areas were cleaned with 70% alcohol and allowed to dry. Skin scrapings were collected from the active erythematous margins of the lesions using a sterile blade. Nail clippings were obtained using a sterile nail cutter. Scrapings from the scaly lesions over the scalp or epilated hair, including the roots, were collected using sterile forceps.

Sample processing

The sample collected was treated with 10% potassium hydroxide (KOH) for skin and hair samples and 20% KOH for nail samples and subjected to direct microscopic examination to detect the fungal elements. The samples, irrespective of demonstration of fungal elements by direct microscopic examination, are inoculated on fungal culture media containing Sabouraud dextrose agar (SDA) without chloramphenicol and cycloheximide, Sabouraud dextrose agar (SDA) with chloramphenicol and cycloheximide, and dermatophyte test media (DTM) and incubated at 32°C for a period of four weeks.

Based on the growth rate, colony morphology, and pigment production on the culture media, the species was identified. Lactophenol cotton blue preparations (LPCB) were used to detect the presence of macroconidia, microconidia, chlamydospores, and hyphal structures. The culture positivity rates were compared with the direct microscopy findings.

Statistical analysis

The data obtained were entered in a Microsoft Excel sheet (Microsoft Corporation, Redmond, WA, USA), and statistical analysis was performed using JMP® Pro 16 software, version 16 (SAS Institute, Cary, NC, 1989-2021). Results were presented as mean (median) ± SD, counts, percentages, and diagrams. The Pearson chi-square (χ²) test was used for the association between two categorical variables.

## Results

A hospital-based prospective study was conducted from May 2023 to January 2025 at a tertiary care center in Vijayapura. A total of 153 pediatric patients with a clinical diagnosis of dermatophytosis were included in this study.

Children below 15 years of age clinically diagnosed with dermatophytosis were enrolled in the study. The most commonly affected age group was 13-15 years (30.7%). The mean age is nine years with a standard deviation of 4.5 (Table 1).

**Table 1:** Age-wise distribution of patients (age in years)

Age	No. of patients	Percentage
<1 year	8	5.2
1-2 years	6	3.9
3-4 years	22	14.4
5-6 years	14	9.2
7-8 years	14	9.2
9-10 years	18	11.8
11-12 years	24	15.7
13-15 years	47	30.7
Total	153	100.0

A total of 153 patients were enrolled in the study; 111 (72.5%) were males and 42 (27.5%) were females. The male-to-female ratio was 2.65:1 (Table 2).

**Table 2:** Sex distribution of patients in percentage

Gender	No. of patients	Percentage
Female	42	27.5
Male	111	72.5
Total	153	100.0

The majority of affected males belonged to the age group of 13-15 years, with 39 males (35.1%), followed by 11-12 years with 17 males (15.3%). The affected females mostly belonged to the 13-15-year age group, with a total of eight females (19.0%) (Table 3).

**Table 3:** Correlation between sex distribution and age group

Age (Years)	Female	Male	Total
<1 year	3 (7.1%)	5 (4.5%)	8
1-2 years	3 (7.1%)	3 (2.7%)	6
3-4 years	6 (14.3%)	16 (14.4%)	22
5-6 years	5 (11.9%)	9 (8.1%)	14
7-8 years	7 (16.7%)	7 (6.3%)	14
9-10 years	3 (7.1%)	15 (13.5%)	18
11-12 years	7 (16.7%	17 (15.3%)	24
13-15 years	8 (19.0%)	39 (35.1%)	47
Total	42 (100%)	111(100%)	100.0

The duration of the infection varied from two weeks to one year. The majority, 61 patients (39.9%), had an infection for one to two months (Table 4).

**Table 4:** Distribution of patients based on the duration of lesions.

Duration of lesion in months	No. of patients	Percentage
< 1.00	43	28.1
1-2	61	39.9
2-3	21	13.7
3-4	7	4.6
4-5	3	2.0
5-6	0	0
6-7	6	3.9
7-8	0	0
9. +	12	7.8
Total	153	100.0

A history of treatment taken in any form prior to consultation at the tertiary care center was noted in 83 patients (54.2%). Among them, seven patients (4.57%) had a history of taking oral medications along with application of topical medication, while two patients (1.30%) had used only oral medication, and 31 patients (20.26%) had a history of application of topical steroid combination medication alone. A history of taking unknown medication was seen in 43 patients (28.10%) (Table 5).

**Table 5:** Distribution based on the history of previous medications

Type of medication	No. of patients	Percentage
Oral + topical	7	4.57
Oral medication	2	1.30
Topical steroid combination	31	20.26
Unknown medication	43	28.10
Nil	70	45.7
Total	153	100.0

**Figure 1:**
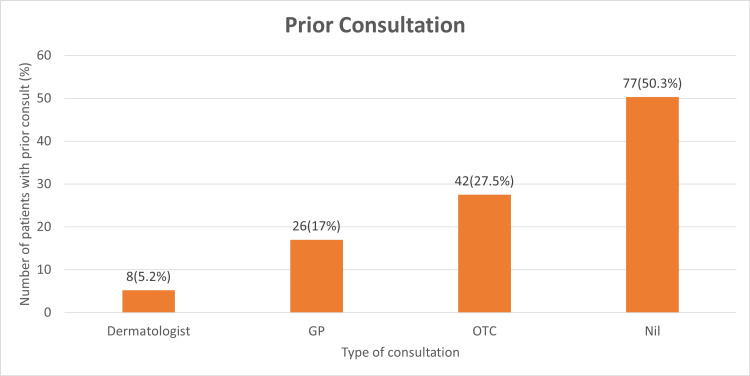
Distribution based on the number of patients who received prior consultation GP: general practicioner, OTC: over the counter

Forty-two patients (27.5%) reported a history of self-treatment with pharmacy-purchased (over-the-counter) medications. Thirty-four patients received treatment after consulting a doctor for their skin disease; 26 of them (17%) consulted a general practitioner, whereas only eight of them (5.2%) had consulted a dermatologist (Figure 1).

History of sharing fomites and sharing of clothes, combs, pillows, and beds among close contacts or family members was habitual among 57 (37.3%) patients (Table 6).

**Table 6:** Distribution of patients based on the sharing of fomites

H/o sharing fomites	No. of patients	Percentage
Present	57	37.3%
Absent	96	62.7%

History of contact with animals (dogs/cats) present in 47 patients (30.7%) (Table 7).

**Table 7:** Distribution based on contact with pets

H/o contact with animals	No. of patients	Percentage
Present	47	30.7
Absent	106	69.3

The majority of the patients had a daily bathing practice (65.4%), and 34.6% of patients had average to poor bathing practices (Table 8).

**Table 8:** Bathing practices of the patients

Bathing practices	No. of patients	Percentage (%)
Everyday	100	65.4
Alternate day	32	20.9
≥2 days	21	13.7
Total	153	100

The most common cutaneous site of involvement was found to be the scalp (24.2%), followed by the groin area (23.5%), while the least common site overall was found to be the nails (Table 9).

**Table 9:** Distribution of patients based on the site of tinea lesion

Site of the presence of lesion	No. of patients	Percentage
Scalp	37	24.2
Face	23	15
Neck	9	5.9
Chest	5	3.3
Back	13	8.5
Abdomen	22	14.4
Upper limb (except hands)	26	17.0
Lower limb (except feet)	22	14.4
Gluteal region	30	19.6
Groin	36	23.5
Dorsum of feet	2	1.3
Dorsum of hands	2	1.3
Palms	6	3.9
Soles	5	3.3
Nails	0	0

**Figure 2:**
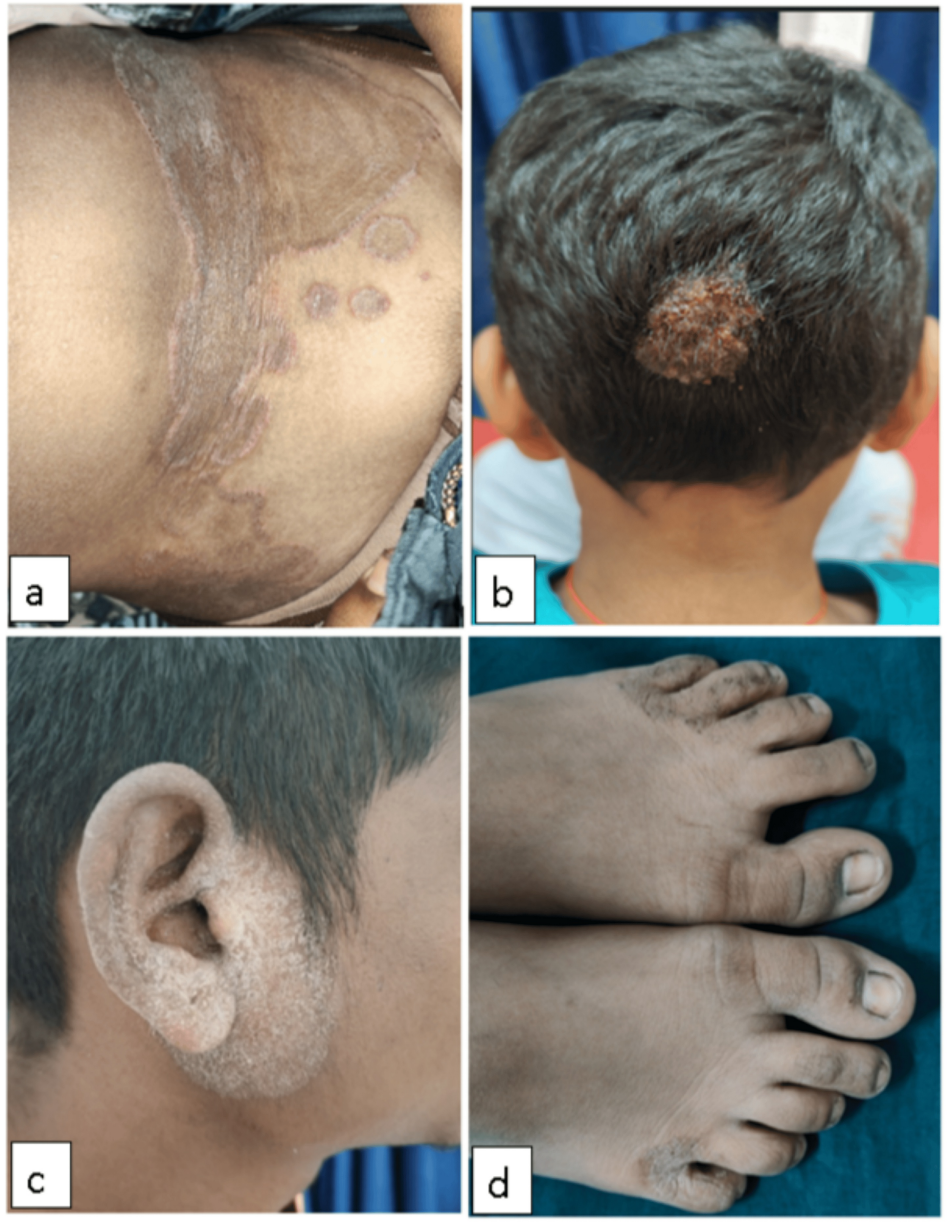
Clinical images showing a) tinea corporis over the abdomen, b) tinea capitis (kerion), c) tinea faciei, and d) tinea pedis in the interdigital space

The most commonly encountered clinical diagnosis was tinea corporis (85.6%), followed by tinea capitis (24.2%), while the least common were tinea manuum (3.9%) and tinea pedis (3.3%) (Figure 2). No cases of tinea unguium (0%) presented during the study period. There were patients with more than one clinical condition (Table 10).

**Table 10:** Distribution of patients according to clinical diagnosis

Examination	No. of patients	Percentage
Tinea capitis	37	24.2
Tinea faciei	23	15.0
Tinea corporis	131	85.6
Tinea cruris	36	23.5
Tinea manuum	6	3.9
Tinea pedis	5	3.3
Tinea unguium	0	0

The most frequently seen mixed type of clinical diagnosis was tinea corporis along with tinea cruris, present in 23 patients (15.2%), followed by tinea capitis along with tinea corporis, seen in four patients (2.6%) (Table 11).

**Table 11:** Distribution of patients based on a mixed type of dermatophytosis.

Clinical diagnosis	No. of patients
		T. corporis + T. cruris	23
T. capitis + T. corporis	4
T. capitis + T. corporis + T. cruris	1
T. capitis + T. cruris + T. corporis + T. faciei	1
T. corporis + T. cruris + T. pedis	1
T. corporis + T. cruris + T. pedis + T. manuum	2
T. corporis + T. faciei	3
T. corporis + T. manuum	1
T. corporis + T. manuum + T. pedis	1
T. curis + T. corporis + T. faciei	1
Total	38

**Figure 3:**
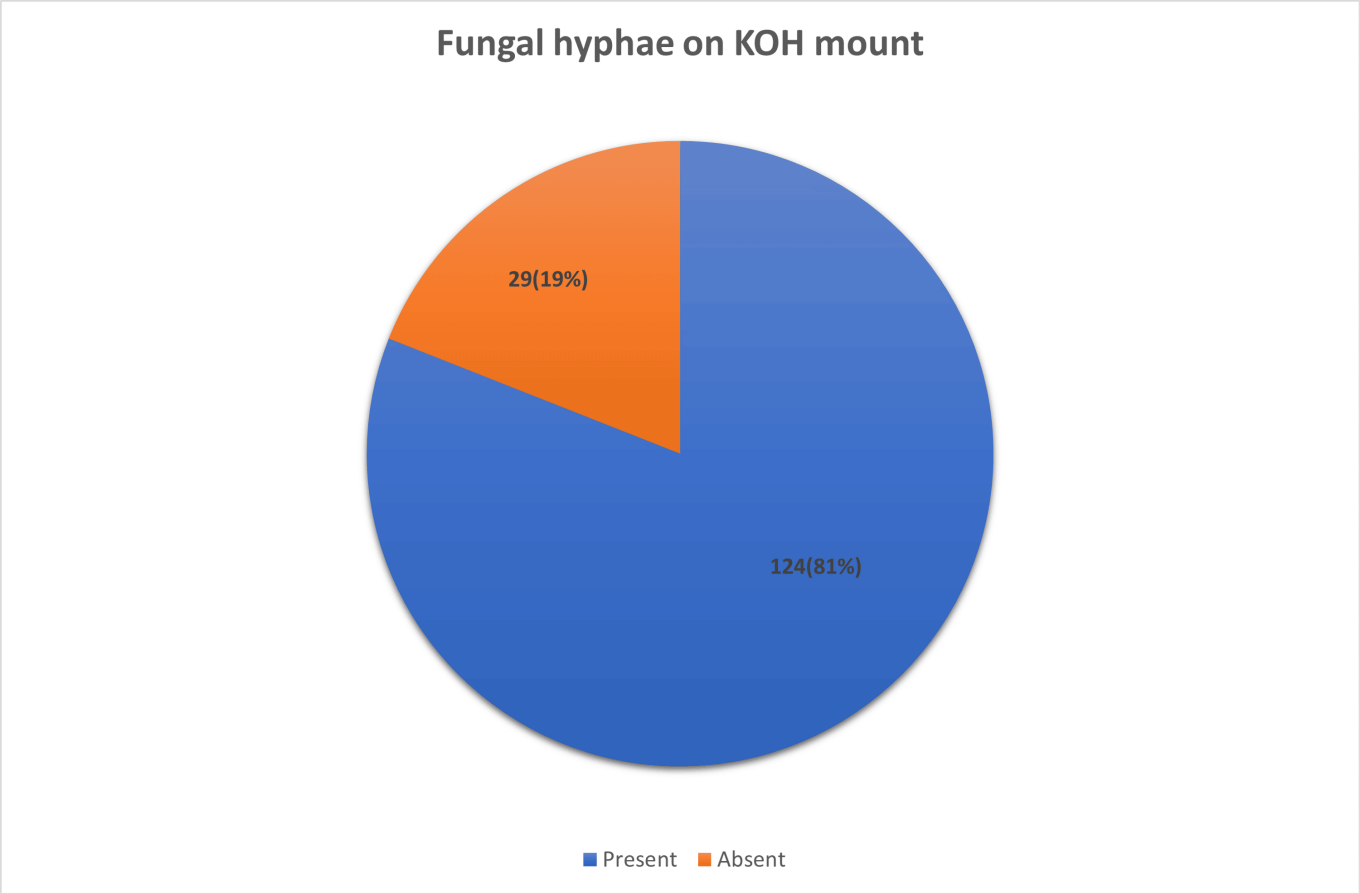
Number of patient samples with the presence of fungal hyphae on the direct microscopy of the potassium hydroxide (KOH) mount.

Examination of skin scrapings with a 10% KOH solution showed the presence of fungal hyphae in 124 patients (81%) (Figure 3).

The correlation between the KOH mount and fungal culture was analyzed, and it was found that both microscopy and culture were positive in 73 samples (58.9%), while 51 (41.1%) samples were positive by microscopy but negative by culture. Although negative by microscopy, culture positivity was seen in five (17.2%) samples, and both negative results were evident in 24 cases (82.8%) (χ² = 16.299, p = 0.001) (Table 12).

**Table 12:** Correlation between the presence of fungal hyphae in the 10% KOH mount and dermatophytic growth on culture media.

Culture positive for dermatophytes	Fungal hyphae absent in the 10% KOH mount	Fungal hyphae present in the 10% KOH mount
Positive	5 (17.2%)	73 (58.9%)
Negative	24 (82.8%)	51 (41.1%)

Of the 153 samples sent for culture in various media, a positive growth for dermatophytes was seen in 78 samples (51%) (Table 13).

**Table 13:** Total number of samples that had a growth in any one of the fungal culture media

Culture positive for dermatophytes	No. of samples	Percentage
Present	78	51.0
Absent	75	49.0
Total	153	100

Among the media with dermatophytic growth (78), 77 patient samples had growth on dermatophyte test media (98.7%); 63 patient samples had growth on SDA with antibiotics media (80.7%), while 31 patients showed growth on plain SDA media (39.7%) (Table 14).

**Table 14:** Dermatophytic growth on the culture media SDA: Sabouraud dextrose agar

Dermatophytic growth on media	No. of samples	Percentage (%)
SDA without antibiotic		
Present	31	39.7
Absent	47	60.25
SDA with cycloheximide and chloramphenicol		
Present	63	80.8
Absent	15	19.23
Dermatophyte test media		
Present	77	98.7
Absent	1	1.28
Total	78	100

**Figure 4:**
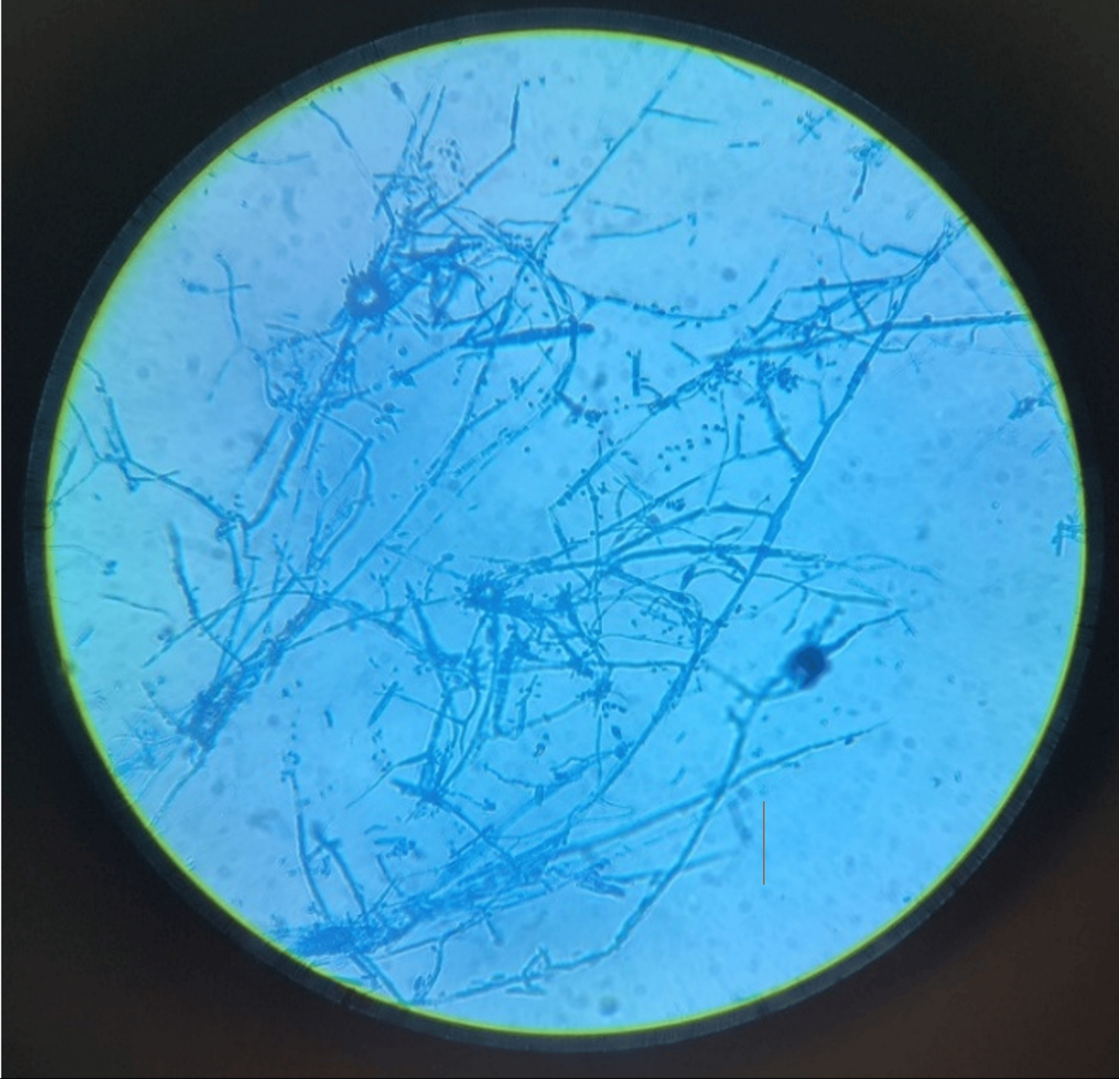
Microscopic morphology of T. mentagrophytes on Lactophenol cotton blue mount-Spiral hyphae with spherical microconidia arranged in cluster.

In this study, species isolation was possible from 78 samples (51%) out of 153 samples collected. Among the 78 samples, *T. mentagrophytes* was isolated from 49 patients (62.8%) (Figure 4). *T. rubrum* from 18 (23.1%), *T. tonsurans* from seven (9%), and *M. canis* from four (5.1%) patients were isolated. *T. mentagrophytes* (62.8%) was noted to be the most commonly isolated species, followed by *T. rubrum* (23.1%) (Table 15).

**Table 15:** Distribution of isolated species of dermatophytes

Organism	No. of samples isolated	Percentage (%)
T. mentagrophytes	49	62.8
T. rubrum	18	23.1
T. tonsurans	7	9
M. canis	4	5.1
Total	78	100.0

## Discussion

Age

The study highlighted that dermatophytosis commonly affected the 13-15-year-old age group (30.7%) and the 11-12-year-old age group (15.7%). This aligns with studies done by Ray A et al. [9] and Dash M et al. [10]. Predisposing factors attributed to rising dermatophytic infection in children are hormonal changes, poverty, overcrowding, more participation in outdoor activities with increased sweating, and negligence of personal hygiene.

Gender

Dermatophyte infection was more commonly seen in males than females in our study, with a sex ratio of 2.65:1. Almost all the studies have reported male predominance [11,12], and except for a few studies, they have shown female predominance [13]. It has been postulated that the low prevalence in females could be associated with the fact that the majority tend to practice better personal and hair hygiene when compared to males [12].

Duration of lesion

The majority of patients had a lesion duration of one to two months. A similar finding was noted by Ray et al. [9] and Mishra et al. [14]. The prolonged duration of the lesion can be ascribed to the patient's negligence, self-medication habit, and limited access to high-quality healthcare facilities. Long duration negatively impacts the child’s quality of life with considerable psychological distress, especially in recurrent cases [15].

Past history

A history of taking treatment of any form prior to consultation at the tertiary care center was observed in 83 patients (54.2%). A history of application of topical steroid combination medication was seen in 20.26%. Most over-the-counter topical drug combinations contain clobetasol propionate in combination with topical antifungals and/or antibacterials. Due to their widespread availability and low price, the community misuses such topical preparations [16].

The majority of patients had a history of self-medication, and only a few patients (5.2%) had consulted a dermatologist prior to consultation at the tertiary care center. This signifies that those who failed to visit a dermatologist were not diagnosed and treated appropriately. The widespread availability of over-the-counter antifungal-steroid combination medications accounts for this.

However, this relief is only temporary, and abuse of the steroid medication increases the risk of developing recalcitrant dermatophytosis [17]. Dash et al. [10] have reported that a majority of their participants (61.11%) were also treated by non-dermatologists and with steroid creams.

In our study, 21.6% of the children had experienced similar problems in the past. This is similar to the observation by Kashyap et al. [18]. According to our findings, the prevalence of recurrent dermatophytosis and steroid-modified tinea in the rural population where this study was conducted is owing to a lack of personal hygiene.

Family history

A history of similar complaints among family members was present in 64 (41.8%) patients. Studies done by Ray et al. [9] and Kashyap et al. [18] showed that 70% and 60% of cases had a history of contact with an infected family member. This is explained by the tradition of family members sharing towels, pillows, and other items. Thus, to prevent recurrences and chronicity of infection, it is imperative to ensure adequate treatment for all affected family members.

Personal history

Furthermore, 34.6% of patients had average to poor personal hygiene practices. This result is similar to the study done by Ray A et al. [9]. Illiteracy, overcrowding, and low socioeconomic conditions can be the reasons for poor personal hygiene. Sharing of clothes, combs, pillows, and beds among family members was habitual among 57 (37.3%) patients. This was explained in a study done by Gupta A et al. [19]. In the background of hot and humid climatic conditions, tightly fitted clothes along the groin, waistline, and infra-mammary area provide a moist and occlusive environment that favors the growth of dermatophytes [17].

A history of contact with animals, such as dogs or cats, was present in 47 patients (30.7%). Contact history with pets is a possible risk factor for dermatophytosis [20].

Clinical examination

On examination, the most common site of cutaneous involvement was the scalp (24.2%), followed by the groin area (23.5%), while the least common site overall was found to nails. These findings resonate with previous studies in which the scalp was the predominant reported site [21,22,23].

Clinical diagnosis

The most common clinical diagnosis was tinea corporis (85.6%), followed by tinea capitis (24.2%). Studies done by Ray A et al. [9], Dash M et al. [10], and Mishra N et al. [14] also showed tinea corporis as the most common clinical diagnosis.

Laboratory investigation

In the current study, overall KOH positivity and culture positivity were 81% and 51%, respectively. Moreover, 58.9% of the cases were both KOH and culture-positive. Satheesh et al. reported 86.7% positivity by KOH and 77.9% positivity by culture. Overall culture positivity was 62.1%, and culture positivity for dermatophytosis was 51%. Non-dermatophytic growth included *Aspergillus* and *Candida* species. Inadequate sample collection, bacterial contamination during sample collection, and prior usage of topical antifungal medication might be the reasons for culture negativity.

The highest dermatophytic growth rate was observed on dermatophyte test media (98.7%), followed by SDA with cycloheximide and chloramphenicol (80.8%). The most commonly isolated species was *T. mentagrophytes* (62.8%), followed by *T. rubrum* (23.1%). Other species isolated included *T. tonsurans* (9%) and *M. canis* (5.1%). These findings align with the findings of previous studies done by Satheesh et al. [11] and Mishra et al. [14]. Contrary to the observation, the predominant species isolated was *T. rubrum*, followed by *T. mentagrophytes* in studies done by Kashyap et al. [18].

Limitations

Our study was limited to a single tertiary care hospital, and it may not reflect the complete mycological picture of other regions of the nation. The absence of a molecular study (PCR-based sequencing) represents a significant limitation. Omission of antifungal susceptibility testing constitutes a substantial limitation.

## Conclusions

According to our study, tinea corporis was the most common clinical presentation of dermatophytosis, followed by tinea capitis. Tinea corporis, along with tinea cruris, was the common pattern among the combination types. The predominant causative fungal species isolated was *Trichophyton mentagrophytes*, followed by *Trichophyton rubrum*. Our study highlights a shift in the clinical presentation of dermatophytosis among children, characterized by larger and more extensive lesions.
